# No Increased Injury Risk on Artificial Turf in Finnish Premier Division Football

**DOI:** 10.1097/JSM.0000000000001296

**Published:** 2024-11-01

**Authors:** Ville Immonen, Einari Kurittu, Ilari Kuitunen, Tommi Vasankari, Mari Leppänen

**Affiliations:** * The Finnish Football Association, Helsinki, Finland;; † University of Eastern Finland, Institute of Clinical Medicine, Kuopio, Finland;; ‡ University of Tampere, Tampere, Finland;; § Tampere Research Center of Sports Medicine, UKK Institute, Tampere, Finland; and; ¶ UKK Institute, Tampere, Finland.

**Keywords:** football, injury, incidence, artificial turf, playing surface, sports medicine

## Abstract

**Objective:**

To compare the injury risk between natural grass and artificial turf in the male Finnish premier division (Veikkausliiga) matches during the 2019 league season.

**Design:**

A retrospective reanalysis of prospectively collected season-long injury surveillance cohort study.

**Setting:**

Finnish elite-level male football.

**Participants:**

All 12 teams of Veikkausliiga participated in the study. All players were eligible to participate. Two hundred thirty-six players took part in the follow-up between February and November 2019.

**Interventions:**

Matches were played between April and November 2019. Injury data were collected using standard injury reports from the team medicals and weekly player questionnaires. Individual player exposure in matches was collected. Artificial turf was classified as exposure and natural grass as control in the analyses.

**Main Outcome Measures:**

Match-related injury incidence and incidence rate ratios (IRR) with 95% confidence intervals (CI) on natural grass and artificial turf.

**Results:**

A total of 113 injuries (65 on artificial turf, 48 on natural grass) in 167 matches (90 on artificial turf, 77 on natural grass) were included. Injury incidence was 27.2/1000 hours on artificial turf and 23.9/1000 hours on natural grass and (IRR, 1.1; 95% CI, 0.8–1.7). There was no difference in injury types, anatomical regions, injury recurrence, injury severity, or injury contact. Forwards may have had an increased injury incidence on artificial turf compared with natural grass (IRR, 3.4; 95% CI, 1.0–11.8).

**Conclusions:**

This study supports previous reports that there is no significant increase in the overall risk of injury in professional football played on artificial turf compared with that on natural grass.

## Abstract 2

Supplemental Digital Content is Available in the Text.

## INTRODUCTION

Professional football has been traditionally played on natural grass (NG). Artificial turfs (AT) have been an option as a playing surface since the 1960s and are nowadays also used in professional football especially in colder regions such as northern Europe. Compared with natural grass, artificial turf provides multiple advantages such as better weather resistance and easier maintenance. In questionnaires, professional footballers have had relatively negative perceptions toward artificial turf. One reason for this might be the fact that players' experiences and football actions are known to be different on artificial turf.^1–3^ It is also known that professional players consider artificial turf as a major injury factor in football.^4,5^

Studies made in the 1990s indicated that artificial turf might significantly increase injury risk compared with natural grass.^6^ However, artificial materials have developed widely during the 21st century. Most recent studies that have compared overall acute injury incidence in professional football matches (men and/or women) between natural grass and modern third generation artificial turfs have either found no difference^7–12^ or even lower overall injury incidence on artificial turf.^13^ Some studies have indicated increase in the incidence of specific injury types or locations on artificial turf^8,9,12,14^ but not the overall injury incidence. The most recent meta-analysis including 22 cohort studies showed a decrease in overall injury incidence on artificial turf compared with natural grass,^15^ however, training injuries and amateur players were also included. Studies show that injury risk on artificial turf is most likely dependent on the sport. For example, fewer concussions on artificial turf have been reported in American football and rugby, while in football, no clear evidence has been found.^16^

In most parts of the world, artificial turf is still used very little in professional football that limits the research possibilities. Relatively quite a few cohort studies have been made in Sweden and Norway earlier this century both in professional^10,11,17^ and amateur football.^18–20^ In these studies, most of the professional teams used natural grass as their primary surface opposed to this study. Also, as mentioned earlier, artificial materials are still developing constantly and updated research of the subject is needed. There are no previous studies that compare injury incidence between different playing surfaces in Finnish professional football. Injury incidence and prevalence rates in Finnish professional football have previously been analyzed, but this study did not report incidences stratified by playing surface.^21^

The aim of this study was to compare the injury incidence between natural grass and artificial turf in the male Finnish premier division matches during the 2019 league season.

## METHODS

### Study Design

This investigation is a part of a prospective 1-season cohort study among male football players in Finland.^21^ The current study is an additional analysis investigating the effect of playing surface on injury risk.

### Study Population

All 12 teams of Veikkausliiga (the Finnish premier division of male football) were invited to the original study and all teams agreed to participate. Four teams played all of their league home matches on natural grass and 6 teams on artificial turf. Two teams started the season on artificial turf and later changed to natural grass as soon as their home pitch was in decent condition.

Every player who was an official member of a participating team was eligible to be included in the study. Players and the guardians of underaged players (a total of 14 players) were informed about the study in December 2018. A written informed consent was required for a player to be included.

### Data Collection

The injury data were collected between February and November 2019 (10-month follow-up) and are described in detail elsewhere.^21^ In the 2019 season, league matches in the Finnish premier division were played between April 3 and November 3. At the beginning of the study, players completed a questionnaire that included basic demographic information, playing position, playing history, and previous injuries. Age and playing history were reported by all 236 included players, while player position and injury/health history were reported by 194 included players.

Match injury data during the season were collected using standard injury report from the team medical staff. This report included the injury date, type, location, diagnosis, mechanism (contact/noncontact), and return date. The report forms were sent to researchers at the end of the season as soon as all the information was collected. In addition to the report forms, the original data were completed with weekly player health questionnaires; however, this information was not used in this study.

An injury was defined as any physical complaint sustained by a player during football match play, irrespective of the need for medical attention or time loss from football activities. An acute injury was defined as an injury result from a single, specific, identifiable event. Injury severity was based on medical staff reports and reported as days of absence from full participation in team training and playing. Severity of injuries was categorized as slight (0 days, similar to nontime-loss injury), mild (1–7 days), moderate (8–28 days), and severe (>28 days). Overuse injuries were excluded from this analysis. Recurrent injury was defined as injury of the same type at the same location as the original injury, occurring only after player's return to full participation between the injuries.

In the original material, match events were categorized to league, training, national team, cup, youth, reserve, and undefined matches. All reported league match injuries were initially included in this study. If the match event information was missing or the injury was reported as an undefined match injury, it was presumed to be a league match injury if the injured players' team played a league match on the same date.

Individual player exposure in matches was collected each week using the weekly questionnaire (QuestBack). In case of missing information, the data were supplemented with reports from medical staff or team websites. Individual exposures on both surfaces were calculated from which overall and positional surface exposures were summarized. In this study, player positions were categorized to forwards (including strikers and wingers), midfielders, defenders (including center backs and full-backs), and goalkeepers.

Injuries were categorized based on the recommendation by the International Olympic Committee.^22^ Injury type categories in this study were muscle/tendon strains, joint/ligament sprains, fractions/contusions, and other injuries (nervous system, skin, cartilage, etc). Anatomical regions of injuries were classified to head and neck, upper limb, trunk, and lower limb injuries. Lower limb injuries were furthermore divided to more specific regions (hip/groin, thigh, knee, lower leg, ankle/foot).

The surface of the injury was gathered from the player reports or team medical reports. If the playing surface information was missing, data were completed based on the match schedule and stadium surface information if the injury date was known. If both the injury date and the playing surface information were missing, the injury was excluded from the study. The Finnish Premier division requires its teams to fulfill the playing surface requirements of the Internation Federation of Associaton Football (FIFA) Quality Concept for Football Turf or FIFA Quality Pro Standard.^23,24^ This means that all the artificial turfs are standardized third generation surfaces, which include both sand and rubber infill.

### Statistical Analysis

Continuous data were reported as mean and standard deviations. Categorized variables were presented as absolute numbers and proportions. Injury incidence was calculated per 1000 hours of exposure with 95% confidence intervals (CI). Incidence rate ratios (IRR) were used to compare artificial turf with natural grass. We used Poisson exact method to calculate CI. Subgroup analyses were performed to examine injury type and anatomical region of injury. We used stratified analyses (injury history, playing position, etc.) as based on directed acyclic graph, player attributable factors cannot be considered as confounders (see **Figure**, **Supplemental Digital Content 1**, http://links.lww.com/JSM/A461).

This study has been reported according to the Strengthening of Reporting in Observational Studies guideline and the checklist is provided as a supplement.

### Ethical Review

The study was approved by the Ethics Committee of Pirkanmaa Hospital District, Tampere, Finland (code R18187). All participating players gave their written informed consent before study onset.

### Data Availability

The data that support the findings of this study are available on request from the corresponding author. The data are not publicly available because of information that could compromise the privacy of research participants.

## RESULTS

A total of 236 players participated in the study. Player’s mean age was 23.8 years and participants had averagely played 3.5 years in the Finnish premier division. All playing positions were represented among participants. Eighty-three players (35.2%) reported they had had a previous orthopedic operation (Table 1).

**TABLE 1.** Player Characteristics (n = 236)

	Mean	SD
Age	23.8	5.1
How many seasons in the Finnish League including the current season during study	3.5	1.9

	n	%
Position		
Striker	28	11.9
Winger	25	10.6
Midfielder	48	20.3
Full back	31	13.1
Center back	36	15.3
Goalkeeper	26	11.0
Not reported	42	17.8
Has had previous orthopedic operation		
Yes	83	35.2
No	111	47.0
Not reported	42	17.8

In the 2019 Veikkausliiga season, 77 matches were played on natural grass and 90 matches on artificial turf (including regular season, postseason, and qualifiers to Eurogames). A total of 4398 hours of match exposure were recorded, of which 2011 hours were on natural grass and 2387 hours on artificial turf.

Originally, 87 league match injuries were reported by the players and team medicals. In addition, 27 injuries that were either missing the match event information or that were reported as undefined match injuries were included based on the match schedule. The playing surface information was added for 25 injuries. One league match injury was excluded for missing both the injury date and the playing surface information. Eventually, a total of 113 injuries (65 on AT, 48 on NG) were included in the study. Forty-three players suffered 1 league match injury and 29 players suffered more than 1 league match injury during the season. Overall injury incidence in league matches was 27.2/1000 hours on artificial turf and 23.9/1000 hours on natural grass (IRR, 1.1; 95% CI, 0.8–1.7; Table 2).

**TABLE 2.** Number of Injuries (n) and Injury Incidence per 1000 h of Exposure on Natural Grass and Artificial Turf

	Artificial Turf		Natural Grass		IRR (95% CI)
	n	Incidence (95% CI)	n	Incidence (95% CI)	
All injuries	65	27.2 (21.2–34.5)	48	23.9 (17.8–31.4)	1.1 (0.8–1.7)
Injury type					
Strain (muscle/tendon)	32	13.4 (9.3–18.7)	18	9.0 (5.5–13.8)	1.5 (0.8–2.7)
Lower limb	31	13.0 (9.0–18.2)	16	8.0 (4.7–12.6)	1.6 (0.9–3.0)
Other	1	0.4 (0.0–2.0)	2	1.0 (0.2–3.2)	0.4 (0.0–4.6)
Sprain (joint/ligament)	8	3.4 (1.6–6.3)	8	4.0 (1.9–7.5)	0.8 (0.3–2.2)
Lower limb	8	3.4 (1.6–6.3)	6	3.0 (1.2–6.2)	1.1 (0.4–3.2)
Other	0	—	2	1.0 (0.2–3.2)	—
Fractures and contusions	15	6.3 (3.7–10.1)	15	7.5 (4.4–12.0)	0.8 (0.4–1.7)
Other	4	1.7 (0.6–4.0)	3	1.5 (0.4–4.0)	1.1 (0.3–5.0)
Undefined or missing information	6		4		
Anatomical region					
Head and neck	6	2.5 (1.0–5.2)	6	3.0 (1.2–6.2)	0.8 (0.3–2.6)
Upper limb	0	—	4	2.0 (0.7–4.7)	—
Trunk	2	0.8 (0.2–2.7)	4	2.0 (0.7–4.7)	0.4 (0.1–2.3)
Lower limb	57	23.9 (18.2–30.7)	34	16.9 (11.9–23.3)	1.4 (0.9–2.2)
Hip/groin	11	4.6 (2.5–8.0)	7	3.5 (1.6–6.8)	1.3 (0.5–3.4)
Thigh	23	9.6 (6.3–14.2)	12	6.0 (3.3–10.1)	1.6 (0.8–3.2)
Knee	7	2.9 (1.3–5.8)	5	2.5 (0.9–5.5)	1.2 (0.4–3.7)
Lower leg	6	2.5 (1.0–5.2)	6	3.0 (1.2–6.2)	0.8 (0.3–2.6)
Ankle/foot	10	4.2 (2.2–7.4)	4	2.0 (0.7–4.7)	2.1 (0.7–6.7)
Injury recurrence					
Yes	6	2.5 (1.0–5.2)	4	2.0 (0.7–4.7)	1.3 (0.4–4.5)
No	49	20.5 (15.4–26.9)	34	16.9 (11.9–23.3)	1.2 (0.8–1.9)
Missing information	10		10		
Contact					
Direct contact injury	27	11.3 (7.6–16.2)	28	13.9 (9.5–19.8)	0.8 (0.5–1.4)
Noncontact injury	22	9.2 (5.9–13.7)	10	5.0 (2.6–8.8)	1.9 (0.9–3.9)
Missing information	16		10		
Severity					
Slight (0 d)	4	1.7 (0.6–4.0)	3	1.5 (0.4–4.0)	1.1 (0.3–5.0)
Mild (1–7 d)	22	9.2 (5.9–13.7)	22	10.9 (7.1–16.3)	0.8 (0.5–1.5)
Moderate (8–28 d)	23	9.6 (6.3–14.2)	16	8.0 (4.7–12.6)	1.2 (0.6–2.3)
Severe (>28 d)	12	5.0 (2.7–8.5)	6	3.0 (1.2–6.2)	1.7 (0.6–4.5)
Missing information	4		1		
Previous operation made					
Yes	31	13.0 (9.0–18.2)	23	11.4 (7.4–16.9)	1.1 (0.7–2.0)
No	29	12.2 (8.3–17.2)	18	9.0 (5.5–13.8)	1.4 (0.8–2.4)
Missing information	5		7		

Comparisons made by incidence rate ratios with 95% CI.

The most common injury type was muscle/tendon strain. The incidence of all strain injuries was 13.4/1000 hours on artificial turf and 9.0/1000 hours on natural grass (IRR, 1.5; 95% CI, 0.8–2.7). Vast majority of strain injuries occurred in the lower limb area (13.0/1000 hours on AT vs 8.0/1000 hours on NG; IRR, 1.6; 95% CI, 0.9–3.0). There was no difference in injury types between different surfaces. Injury type information was undefined or missing from 10 reported injuries (Table 2).

Anatomically, 81% of injuries occurred in the lower limb area. The incidence of lower limb injuries was 23.9/1000 hours on artificial turf and 16.9/1000 hours on natural grass (IRR, 1.4; 95% CI, 0.9–2.2). There was no evidence of a difference in anatomical main categories or lower limb injury subgroups (Table 2).

Four individual players suffered a total of 6 recurrent injuries in league matches. Incidence of injury recurrency was 2.5/1000 hours on artificial turf and 2.0/1000 hours on natural grass (IRR, 1.3; 95% CI, 0.4–4.5). There was no evidence of a difference in injury recurrence rate between the surfaces; however, this information was missing from 10 injuries on both surfaces (Table 2).

Direct contact injury incidence was 11.3/1000 hours artificial turf and 13.9/1000 hours on natural grass (IRR, 0.8; 95% CI, 0.5–1.4). Noncontact injury incidence was 9.2/1000 hours on artificial turf and 5.0/1000 hours on natural grass (IRR, 1.9; 95% CI, 0.9–3.9). There was no evidence of a difference in direct contact or noncontact injuries between surfaces. The injury contact information was either missing or uncertain in 16 injuries on artificial turf and 10 injuries on natural grass (Table 2).

Most reported injuries were either mild (1–7 days absence) or moderate (8–28 days absence) on both artificial turf and natural grass. Incidence of mild severity injuries was 9.2/1000 hours on artificial turf and 10.9/1000 hours on natural grass (IRR, 0.8; 95% CI, 0.5–1.5), and incidence of moderate severity injuries was 9.6/1000 hours on artificial turf and 8.0/1000 hours on natural grass (IRR, 1.2; 95% CI, 0.6–2.3). There was no significant difference in injury severity in these categories between the surfaces. Also, no significant difference was found in nontime-loss injuries (1.7/1000 hours on AT vs 1.5/1000 hours on NG; IRR, 1.1; 95% CI, 0.3–5.0) or severe injuries (5.0/1000 hours on AT vs 3.0/1000 hours on NG, IRR, 1.7; 95% CI, 0.6–4.5). Severity information was missing from 4 injuries on artificial turf and 1 injury on natural grass (Table 2).

Players who reported that a previous orthopedic operation had been made to them had similar injury incidence on artificial turf (13.0/1000 hours) and natural grass (11.4/1000 hours, IRR, 1.1; 95% CI, 0.7–2.0). There was also no difference in players who had not been operated previously (12.2/1000 hours on AT vs 9.0/1000 hours on NG, IRR, 1.4; 95% CI, 0.8–2.4) (Table 2).

Player position was known for 194 of 236 players. Defenders had the highest injury incidence (40.4/1000 hours) on artificial turf and midfielders (37.4/1000 hours) on natural grass. In the forward position, injury incidence was 33.8/1000 hours on artificial turf and 9.9/1000 hours on natural grass (IRR, 3.4; 95% CI, 1.0–11.8). There was no difference in other positions between the surfaces (Table 3).

**TABLE 3.** Number of Injuries (n) and Injury Incidence in Player Positions per 1000 h of Exposure on Natural Grass and Artificial Turf

Position (N = No. of Players)	Artificial Turf		Natural Grass		IRR (95% CI)
	n	Incidence (95% CI)	n	Incidence (95% CI)	
Striker (N = 53)	15	33.8 (19.7–54.3)	3	9.9 (2.7–26.5)	3.4 (1.0–11.8)
Midfielder (N = 48)	15	30.6 (17.9–49.2)	17	37.4 (22.6–58.5)	0.8 (0.4–1.6)
Defender (N = 67)	29	40.4 (27.6–57.2)	19	33.4 (20.8–51.2)	1.2 (0.7–2.2)
Goalkeeper (N = 26)	1	5.2 (0.5–24.2)	2	12.9 (2.6–41.4)	0.4 (0.0–4.4)

Comparisons made by incidence rate ratios with 95% CI.

## DISCUSSION

In this study population, even though the overall injury incidence was slightly higher on artificial turf than on natural grass, the difference was not statistically significant.

There was no clear difference between injury types. Earlier, few comparable studies have reported a decreased incidence of joint/ligament sprain injuries on artificial turf.^9,15^ In this population, there were slightly more muscle/tendon strain injuries on artificial turf but the result was not statistically significant.

Lower limb injuries were more frequent on artificial turf, but because of wide CI, the result is not significant. Few studies have previously found higher injury rates in ankle injuries on artificial turf,^8,9,12^ but a single study reporting youth injuries has also found lower ankle injury rates on artificial turf.^19^ There were relatively more ankle/foot injuries on artificial turf in this study, but the result is not statistically significant possibly because of low number of injuries.

There was no difference in injury recurrence rate between surfaces. Number of direct contact injuries between surfaces was very similar, but the number of noncontact injuries was relatively higher on artificial turf. However, the result is not statistically significant and contact information was also missing from multiple included injuries. Recent meta-analysis containing 6 studies found noncontact injury incidence on artificial turf lower than that on natural grass in football,^15^ although it is notable that youth, amateur, and training injuries were included.

Also, no difference in injury severity was found in this study. Previous studies have found injury severity on artificial turf either similar^8,9,11,25^ or lower^13,26,27^ than that on natural grass in professional football. Players who had had a previous orthopedic operation also had similar injury incidence between the surfaces; however, it is notable that the cause and location of operation were not specified in this study.

In position comparison, forwards sustained less injuries than midfielders and defenders on natural grass, but on artificial turf, the incidences were quite similar. Although the difference did not quite reach statistical significance, this study may indicate a higher injury risk on artificial turf than on natural grass in forward position. However, no clear conclusions can be made based on this border line finding. Previously in youth football, there have been indications that strikers may have lower injury incidence than defenders,^28^ but the effect of playing surface to positional injuries was not considered.

To our knowledge, this is the first study comparing injury incidence between artificial turf and natural grass in Finland. The results support the recent similar studies made in other Nordic countries.^10,11,19^ The 2019 Veikkausliiga season is so far the last season when match exposure between artificial turf and natural grass in the Finnish League was close to even. In the following 2024 Veikkausliiga season, there are only 2 teams left that have natural grass as their home stadium surface.

This study has some limitations. The number of injuries especially in subgroups was relatively low that causes imprecision to the estimates. Also, the climatic differences between cities and seasons were not taken into account. Artificial turf is generally used more in the northern parts of Finland where the average temperature is lower. Nationally, average temperature varies throughout the season from approximately 3 to 15°C.^29^ We did not find previous articles about the effect of cold weather in football injuries, but in American football and rugby, there has been indications that cold weather might actually reduce injury risk.^30,31^ This topic still needs more research but 1 theory is that cold weather reduces shoe–surface traction that would prevent injuries. Also, the average amount of rain varies from 31 to 75 mm per month through the Finnish premier division season.^29^ In a previous study, dry weather and dry surface were associated with higher incidences of noncontact anterior cruciate ligament injuries in football.^32^ These climatic issues narrow the generalizability of this study.

We did not analyze the exposure of training and other match events on different surfaces or the impact of surface shifts during the season, which is another limitation in this study. A previous study showed that teams with artificial turf as their home venue might have higher acute injury incidence in training but not in matches.^10^ In addition, overuse injuries were excluded from the study because all the teams were facing constant surface changes during the season. Previously, no difference between overuse injuries and surface shifts has been found.^17^ It is also notable that this is an observing study where the team medicals self-evaluated the injuries and, therefore, measurement bias can be significant.

The results of this study support the previous assumption that there is no evidence of increased injury risk in artificial turf in professional football. To current knowledge, injuries should not be used as an argument against artificial turf when making decisions about playing surfaces. Studies with larger populations in the areas where both artificial turfs and natural grasses are used in football are needed to gain more reliable results. Future research is also needed as the artificial materials develop and newer generation artificial turfs with biofilling are being installed. The popularity of artificial turf continues to increase and thus continuous surveillance on safety is needed.

## Supplementary Material

SUPPLEMENTARY MATERIAL
