# Supplementary material for: No Increased Injury Risk on Artificial Turf in Finnish Premier Division Football
Source: Clin J Sport Med. 2024 Nov 1;35(6):663–8. doi: 10.1097/JSM.0000000000001296 (PMC12560186; doi:10.1097/JSM.0000000000001296)

**Supplementary Figure 1** Directed acyclic graph examining the causal relationship between exposure (playing surface) and outcome (injury). Based on the assessment no potential confounders were detected. Player attributable factors are estimated via stratification and weather was not recorded to dataset. Residual confounding is possible as not all sources of bias were controlled.


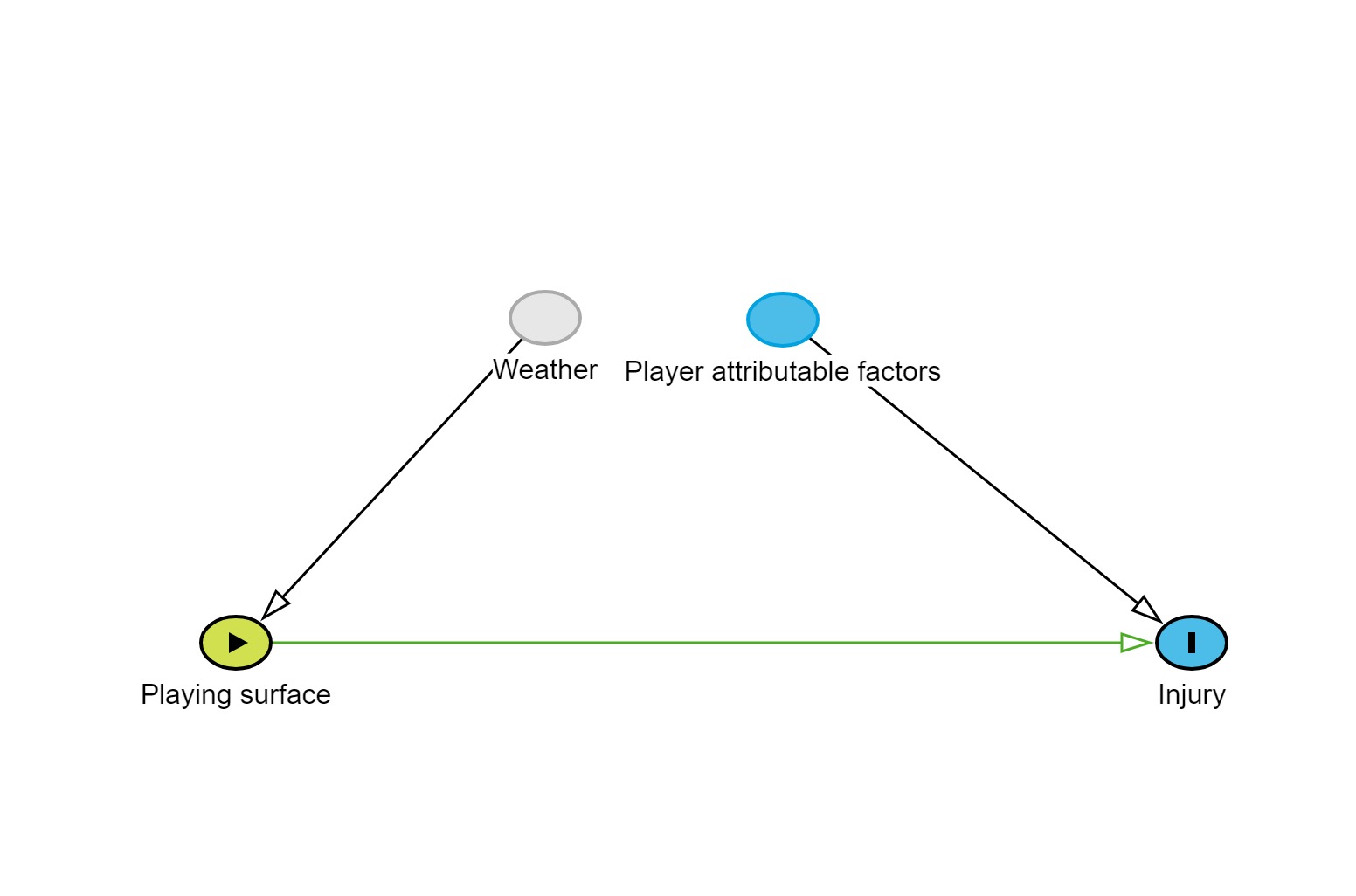

Supplement: SUPPLEMENTARY MATERIAL [file cjsm-35-663-s001.docx]
